# Relationship between Cortical Bone Thickness and Cancellous Bone Density at Dental Implant Sites in the Jawbone

**DOI:** 10.3390/diagnostics10090710

**Published:** 2020-09-17

**Authors:** Shiuan-Hui Wang, Yen-Wen Shen, Lih-Jyh Fuh, Shin-Lei Peng, Ming-Tzu Tsai, Heng-Li Huang, Jui-Ting Hsu

**Affiliations:** 1 Master Program for Biomedical Engineering, China Medical University, Taichung 404, Taiwan; u104020415@cmu.edu.tw; 2 School of Dentistry, China Medical University, Taichung 404, Taiwan; a2312830@ms28.hinet.net (Y.-W.S.); ljfuh@mail.cmu.edu.tw (L.-J.F.); hlhuang@mail.cmu.edu.tw (H.-L.H.); 3 Department of Dentistry, China Medical University and Hospital, Taichung 404, Taiwan; 4 Department of Biomedical Imaging and Radiological Science, China Medical University, Taichung 404, Taiwan; speng@mail.cmu.edu.tw; 5 Department of Biomedical Engineering, Hungkuang University, Taichung 433, Taiwan; anniemtt@hk.edu.tw; 6 Department of Bioinformatics and Medical Engineering, Asia University, Taichung 413, Taiwan

**Keywords:** jawbone, cancellous bone density, cortical bone thickness, dental cone-beam computed tomography, dental implant site

## Abstract

Dental implant surgery is a common treatment for missing teeth. Its survival rate is considerably affected by host bone quality and quantity, which is often assessed prior to surgery through dental cone-beam computed tomography (CBCT). Dental CBCT was used in this study to evaluate dental implant sites for (1) differences in and (2) correlations between cancellous bone density and cortical bone thickness among four regions of the jawbone. In total, 315 dental implant sites (39 in the anterior mandible, 42 in the anterior maxilla, 107 in the posterior mandible, and 127 in the posterior maxilla) were identified in dental CBCT images from 128 patients. All CBCT images were loaded into Mimics 15.0 to measure cancellous bone density (unit: grayscale value (GV) and cortical bone thickness (unit: mm)). Differences among the four regions of the jawbone were evaluated using one-way analysis of variance and Scheffe’s posttest. Pearson coefficients for correlations between cancellous bone density and cortical bone thickness were also calculated for the four jawbone regions. The results revealed that the mean cancellous bone density was highest in the anterior mandible (722 ± 227 GV), followed by the anterior maxilla (542 ± 208 GV), posterior mandible (535 ± 206 GV), and posterior maxilla (388 ± 206 GV). Cortical bone thickness was highest in the posterior mandible (1.15 ± 0.42 mm), followed by the anterior mandible (1.01 ± 0.32 mm), anterior maxilla (0.89 ± 0.26 mm), and posterior maxilla (0.72 ± 0.19 mm). In the whole jawbone, a weak correlation (*r* = 0.133, *p* = 0.041) was detected between cancellous bone density and cortical bone thickness. Furthermore, except for the anterior maxilla (*r* = 0.306, *p* = 0.048), no correlation between the two bone parameters was observed (all *p* > 0.05). Cancellous bone density and cortical bone thickness varies by implant site in the four regions of the jawbone. The cortical and cancellous bone of a jawbone dental implant site should be evaluated individually before surgery.

## 1. Introduction

Dental implant surgery is a common treatment for missing teeth [1,2]. Its survival rate is closely related to osseointegration ability in patients. Specifically, dental implants with higher initial stability have more favorable osseointegration and are associated with a higher survival rate [3,4,5,6,7]; accordingly, examining the bone condition of a dental implant site in the jawbone is crucial. Generally, jawbone condition is assessed by measuring cancellous bone density and cortical bone thickness.

Numerous studies in dentistry have assessed jawbone condition by using computed tomography (CT) and dental cone-beam CT (CBCT), and have evaluated cancellous bone density prior to dental implant surgery on the basis of bone radiographic density. For CT and dental CBCT images, bone density at a dental implant site can be expressed as Hounsfield units (HU) and grayscale values (GVs) [8,9,10,11,12], respectively. Bone density is generally higher in the mandible and anterior region than it is in the maxilla and posterior regions [13,14]. Studies on bone density at different dental implant sites using CT and CBCT for assessment have reported the following ranking (in descending order) of bone density in different regions: anterior mandible, anterior maxilla, posterior mandible, and posterior mandible [9,10,11,12,15].

In addition to cancellous bone density, cortical bone thickness in the jawbone affects the initial stability of dental implants [4,16,17,18,19,20]. Miyamoto et al. [7] employed resonance frequency analyses to measure the initial stability of 225 dental implants; their results demonstrated that implant sites with thicker cortical bone had higher initial stability. Song et al. [21] reported that cortical bone thickness measured using dental CBCT at implant sites was highly correlated with the initial stability of dental implants. Hsu et al. [4] conducted artificial bone experiments and demonstrated that cortical bone thickness significantly influenced the initial stability of dental implants. Roze et al. [22] examined jawbone structures by using micro-CT and compared the results with the initial stability of dental implants. They discovered that dental implant stability depended greatly on cortical bone thickness. In brief, studies on cancellous bone density, cortical bone thickness, and the success rate of dental implant surgery have indicated that jawbone condition is highly correlated with the survival rate of dental implant surgery. In 2017, Ko et al. [23] analyzed the cortical bone thickness of 661 dental implant sites in 173 patients by using dental CBCT and reported the following rankings (in descending order) for cortical bone thickness in different regions: posterior mandible, anterior mandible, anterior maxilla, and posterior maxilla. In the same year, Gupta et al. [24] measured cortical bone thickness at dental implant sites by using dental CBCT and obtained a similar result. 

Research has indicated that cancellous bone density and cortical bone thickness at dental implant sites differ among distinct jawbone regions; however, the posterior maxilla has the lowest cancellous bone density and cortical bone thickness. Researchers have examined cancellous bone density [9,10,11,12,15] and cortical bone thickness [23,24] by using CT and dental CBCT; however, few have analyzed the correlation between the two parameters. Therefore, dental CBCT was employed in this study to identify (1) differences in and (2) correlations between cancellous bone density and cortical bone thickness at dental implant sites in different jawbone regions.

## 2. Materials and Methods

### 2.1. Dental CBCT Examinations of Patients and Implant Sites

This study was conducted after receiving ethical approval from the Institutional Review Board of China Medical University Hospital (No. CMUH 108-REC2-083), approval date: 3 July 2019. Dental CBCT images were obtained from 128 patients (66 male patients, age: 54.14 ± 14.40 years (mean ± standard deviation); 62 female patients, age: 52.13 ± 13.73 years) who had received dental implants between August 2018 and March 2020. Dental CBCT was performed using a Promax 3D Max (Planmeca, Helsinki, Finland) with the following technical parameters: 96 kV, 12.5 mA, and a voxel resolution of 150 or 200 µm. In total, 315 dental implant sites (39 in the anterior mandible, 42 in the anterior maxilla, 107 in the posterior mandible, and 127 in the posterior maxilla) were identified in the dental CBCT images.

### 2.2. Measurement of Cancellous Bone Density at Dental Implant Sites

**Figure 1:**
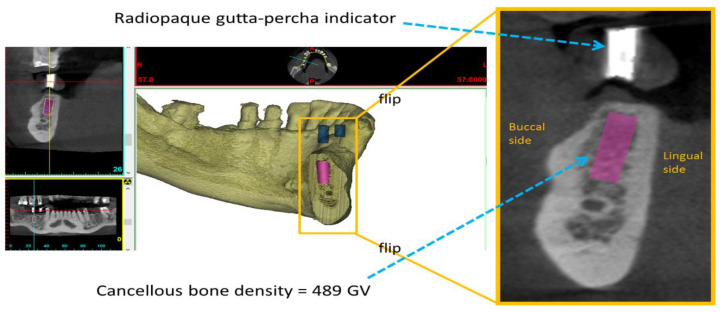
Measurement of cancellous bone density, quantified as the mean grayscale values (GVs) of the cylinder, at a dental implant site.

The CBCT images were loaded into Mimics (Materialise, Leuven, Belgium). Cylinders (3.5–4 mm in diameter and 8–12 mm in length) were virtually defined according to the dental implant site. The size of each cylinder corresponded to the subsequently inserted dental implant. Cancellous bone density was quantified as the mean GV of the cylinder (Figure 1).

### 2.3. Measurement of Cortical Bone Thickness at Dental Implant Sites

**Figure 2:**
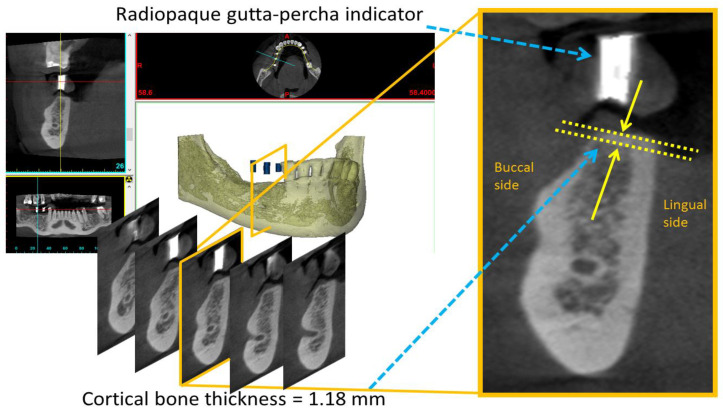
Measurement of cortical bone thickness, distance between the two yellow arrows, at a dental implant site.

In Mimics, continual buccolingual (cross-sectional) images of the maxilla or mandibular bone were captured using the “online reslice” function. To accurately identify the insertion position for a dental implant, diagnostic surgical guide stents with radiopaque gutta-percha indicators were applied for each patient before dental CBCT. Cortical bone thickness was measured in the buccolingual image of the dental implant site, which was made visible by radiopaque gutta-percha indicators (Figure 2).

### 2.4. Statistical Analysis

All statistical analyses were conducted using SPSS Version 19 (IBM Corporation, Armonk, NY, USA) and the significance level was set to *p* < 0.05. The following two statistical methods were used to assess the objectives investigated in this study:

(1) Cancellous bone density or cortical bone thickness differences at dental implant sites in four regions of the jawbone (i.e., the anterior maxilla, posterior maxilla, anterior mandible, and posterior mandible) were analyzed using a one-way analysis of variance (ANOVA) and Scheffe’s post hoc test for multiple comparisons.
(2) The relationship between cancellous bone density and cortical bone thickness at dental implant sites was analyzed using the Pearson correlation coefficients (*r*) for the four regions of the jawbone.

## 3. Results

### 3.1. Cancellous Bone Density and Cortical Bone Thickness at Dental Implant Sites

**Figure 3:**
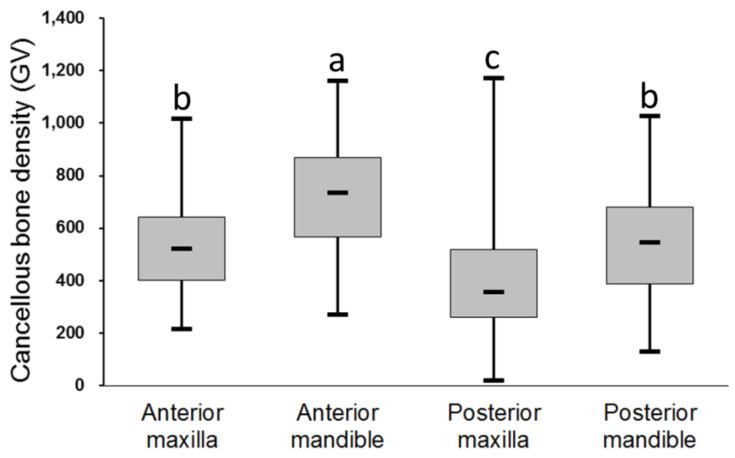
Cancellous bone density at dental implant sites in different regions of the jawbone. Post hoc pairwise comparisons were conducted using Scheffe’s test; use of the same letter indicates no significant difference at the 0.05 level.

For the 315 dental implant sites, cancellous bone density was highest in the anterior mandible (722 ± 227GV) followed by the anterior maxilla (542 ± 208 GV), posterior mandible (535 ± 206 GV), and posterior maxilla (388 ± 206 GV) (Figure 3.). Except for the difference between the posterior mandible and anterior maxilla (*p* = 0.098), the ANOVA and post hoc Scheffe’s test indicated that the cancellous bone density was significantly different among the four regions of the jawbone. 

**Figure 4:**
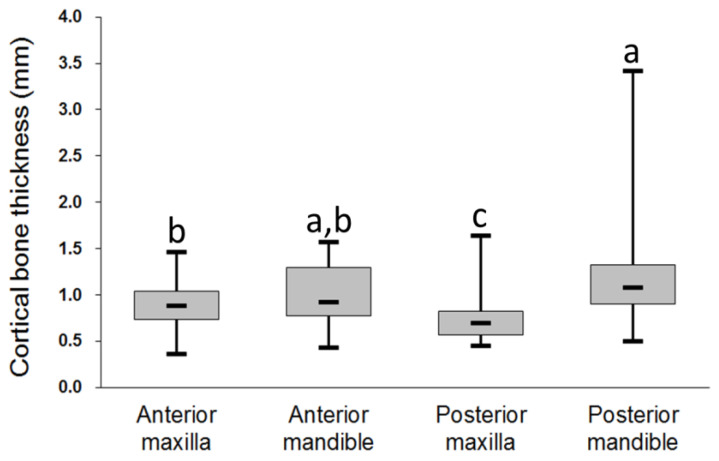
Cortical bone thickness at dental implant sites in different regions of the jawbone. Post hoc pairwise comparisons were conducted using Scheffe’s test; use of the same letter indicates no significant difference at the 0.05 level.

For the 315 dental implant sites, cortical bone thickness was highest in the posterior mandible (1.15 ± 0.42 mm) followed by the anterior mandible (1.01 ± 0.32 mm), anterior maxilla (0.89 ± 0.26 mm), and posterior maxilla (0.72 ± 0.19 mm) (Figure 4). Except for the differences between the anterior maxilla and anterior mandible (*p* = 0.445) and between the anterior mandible and posterior mandible (*p* = 0.110), the ANOVA and post hoc Scheffe’s test indicated that cortical bone thickness differed significantly among the four regions of the jawbone. 

### 3.2. Relationship between Cancellous Bone Density and Cortical Bone Thickness at Dental Implant Sites

For the entire jawbone, a weak correlation between cancellous bone density and cortical bone thickness was detected (*r* = 0.133, *p* = 0.041, Table 1) for the 315 dental implant sites. In addition, except for the maxilla region (*r* = 0.168, *p* = 0.029), posterior region (*r* = 0.178, *p* = 0.006), and anterior maxilla (*r* = 0.306, *p* = 0.048), which all had weak correlations, none of the other regions exhibited correlations between cancellous bone density and cortical bone thickness (Table 1). 

## 4. Discussion

Dental implant surgery has gradually become the prevailing treatment for missing teeth and the assessment of jawbone condition is an essential procedure prior to such surgery. The relevant literature has indicated that a favorable bone condition results in stronger osseointegration and higher dental implant survival rates [25,26]. Currently, cancellous bone density and cortical bone thickness are the most common indicators used to determine bone condition [3,5,6,7,21,22,27]. Researchers have measured the cancellous bone density of jawbones by using CT and dental CBCT [8,9,10,11,12]; however, few have evaluated cortical bone thickness at dental implant sites [23,24], and nearly none have explored the correlation between cortical bone thickness and cancellous bone density at dental implant sites in jawbones. This study was the first to employ dental CBCT to assess patients’ cancellous bone density and cortical bone thickness at dental implant sites and further examine the correlation between the two parameters. Consistent with the relevant literature, this study verified that cancellous bone density and cortical bone thickness differed among distinct jawbone regions. Furthermore, the results indicate that cortical bone thickness and cancellous bone density in the entire jawbone had a weak correlation; of the four jawbone regions, the two indicators exhibited a weak correlation in the anterior maxilla and nonsignificant correlations in the remaining three regions.

Of studies that have examined the relationship between jawbone condition and the success rate of dental implants, Jaffin et al. [5] demonstrated that in the Lekholm and Zarb bone classification [28], the failure rate of dental implants in Type-IV host bones reached 35%, but that in Type-I–III host bones with higher bone quality was only 3%. Jemt et al. [6] also demonstrated that the failure rate of dental implants in high-quality bones was 7.9%, whereas that in low-quality bones was 28.8%. Moreover, clinical literature has disclosed that implant survival rates are higher when implants are embedded in sites with high bone quality, which provide more initial stability for dental implants, according to clinical research [7] and artificial bone experiments [19,29,30]. Higher initial stability contributes to more favorable osseointegration and a higher implant survival rate [3,5,6,31,32]. Accordingly, bone quality and quantity at implant sites can convey crucial information required prior to dental implant surgery. 

Dental CBCT is currently a standard procedure in routine evaluation prior to dental implant surgery. It possesses advantages, such as a low radiation dose and high spatial resolution, and does not create distortion in three-dimensional images or induce image overlapping [33]. Although some researchers have argued that the use of GVs to present dental CBCT results regarding cancellous bone density is imprecise [34,35], other researchers have implied that such a technique is applicable for bone density evaluation because of the maturity of relevant technology [36,37,38,39,40]. Naitoh et al. [39] and Nomura et al. [41] compared CT and dental CBCT in bone density assessment, and both studies concluded that the HU and GV obtained in CT and dental CBCT, respectively, had a strong positive correlation. Parsa et al. [40] compared dental CBCT with CT and micro-CT for measuring bone tissue, and the results indicated that the GV obtained in dental CBCT was highly correlated with the HU and bone volume fraction obtained in CT and micro-CT, respectively. In 2017, Liu et al., [37] indicated that dental CBCT images effectively indicate cancellous bone density and are a suitable instrument for assessment prior to dental implant surgery. Hao et al., [11], David et al. [9], and Naitoh et al. [39] also employed dental CBCT to measure bone density at dental implant sites. 

Numerous researchers have employed CT and dental CBCT to measure cancellous bone density at dental implant sites in jawbones. Of the researchers who have used CT to evaluate the density in the four jawbone regions, Turkyilmaz et al. [12] and de Olivira et al. [15] indicated that bone density differs significantly among regions and is ranked as follows in descending order: anterior mandible, anterior maxilla, posterior mandible, and posterior maxilla. Moreover, Shapurian et al. [14] and Fuh et al. [10] have indicated that the differences among the four jawbone regions are not always statistically significant; nevertheless, the bone density of the mandible and anterior region is generally higher than that of the maxilla and posterior region. Researchers have used dental CBCT as an assessment instrument for bone density at implant sites and reached similar conclusions. According to Hao et al. [11], bone density descended in the following order: anterior mandible (680 ± 142 GV) > anterior maxilla (460 ± 136 GV), posterior mandible (394 ± 128 GV) > posterior maxilla (230 ± 144 GV), which corresponded to the following result of David et al. [9]: anterior mandible (female: 514 ± 243 GV, male: 521 ± 247 GV) > anterior maxilla (female: 354 ± 206 GV, male: 473 ± 208 GV), posterior mandible (female: 234 ± 212 GV, male: 389 ± 220 GV) > posterior maxilla (female: 193 ± 176 GV, male: 250 ± 193 GV). The present study obtained a similar result; cancellous bone density in different regions was ranked as follows in descending order: anterior mandible (722 ± 227 GV), anterior maxilla (542 ± 208 GV), posterior mandible (535 ± 206 GV), and posterior maxilla (388 ± 206 GV). The different dental CBCT models and scanning parameters (e.g., voltage, current, time, and resolution) and the inclusion of patients of a different ethnicity might have contributed to differences in cancellous bone densities between this study and preceding studies [10]. In sum, generally, the results of this and preceding studies demonstrate that cancellous bone density at dental implant sites in jawbones descended in the following order: anterior mandible, anterior maxilla, posterior mandible, and posterior maxilla. 

Previous studies evaluating cortical bone thickness by using dental CBCT have mostly focused on the thickness on the buccal and palatal sides in orthodontic patients [42,43,44]; few have emphasized crestal cortical bone thickness. In 2013, Gerlach et al. [45] employed dental CBCT to evaluate crestal cortical bone thickness at six mandibular dental implant sites in a patient with missing teeth and obtained a thickness of 2.00 ± 0.15 mm. However, crestal cortical bone thickness might be overestimated because of the partial volume effects caused by the 400 µm resolution in dental CBCT. In 2017, Ko et al. [23] adopted dental CBCT with a resolution of 155 µm to evaluate crestal cortical bone thickness at dental implant sites in the four jawbone regions and revealed that thickness was ranked as follows in descending order: posterior mandible (1.22 ± 0.52 mm) > anterior mandible (1.06 ± 0.32 mm) > anterior maxilla (0.83 ± 0.31 mm) > posterior maxilla (0.72 ± 0.29 mm). In the same year, Gupta et al. [24] implemented similar experiments and obtained a similar order of posterior mandible (1.18 ± 0.48 mm) > anterior mandible (1.08 ± 0.30 mm) > anterior maxilla (0.82 ± 0.32 mm) > posterior maxilla (0.76 ± 0.29 mm). The study of Gupta et al. [24] was based in India, and patient ethnicity was mainly Caucasian and Australian. The experimental results of Ko et al., [23] and the present study were similar because patients in both studies had Mongolian ethnicity. In brief, the present and preceding studies have all indicated that crestal cortical bone thickness at dental implant sites is greatest in the posterior mandible, followed by the anterior mandible, anterior maxilla, and posterior maxilla. 

This study analyzed the correlation between cancellous bone density and cortical bone thickness at dental implant sites by using the Pearson correlation coefficient. The statistical results for the different jawbone regions demonstrated that the two parameters had no correlation in the posterior maxilla, anterior mandible, or posterior mandible (*p* > 0.05) but had a weak correlation in the anterior maxilla (*r* = 0.306, *p* = 0.048), with *p* approaching 0.05. Notably, when the jawbones were divided into only anterior and posterior regions, a significant but weak correlation was observed in the posterior region (*r* = 0.178, *p* = 0.006); when the jawbones were classified into only maxilla and mandible regions, a significant but weak correlation was also observed in the maxilla region (*r* = 0.168, *p* = 0.029). Furthermore, the results for the entire jawbone indicate that cancellous bone density and cortical bone thickness had a weak correlation (*r* = 0.133, *p* = 0.041).

Compared with parameters of cancellous bone density, crestal cortical bone thickness parameters in the anterior mandible were not optimal in the four jawbone regions. Crestal cortical bone thickness may have been lower in anterior mandible than in the posterior mandible for the following reasons: (1) The mandible is categorized as a class III lever; to balance mandible and total blood weight under functional demands, the lighter anterior mandible exerts less energy to achieve mandibular movements than does the heavier anterior mandible [46,47]. (2) Anterior teeth tend to withstand more frequent oblique force than do posterior teeth; specifically, because the anterior mandible frequently tolerates horizontal force, it requires high-density cancellous bone to resist the horizontal force transmitted from the roots to the surrounding bones. (3) Masseter muscles are attached to the buccal side of mandible, but jawbone growth increases crestal cortical bone thickness. Although Cassetta et al. [48] measured crestal cortical bone thickness on the lingual and buccal sides, they reported that the cortical bones in the posterior region were thicker than those in the anterior region. In addition, regardless of crestal cortical bone thickness or cancellous bone density, the posterior maxilla had the lowest bone quality and quantity because it is located near the maxillary sinus. Accordingly, tooth loss often results in pneumatization, deteriorates the bone in the posterior maxilla, reduces cancellous bone density, and reduces cortical bone thickness. These reasons correspond with the results of previous studies [23,24]. 

**Figure 5:**
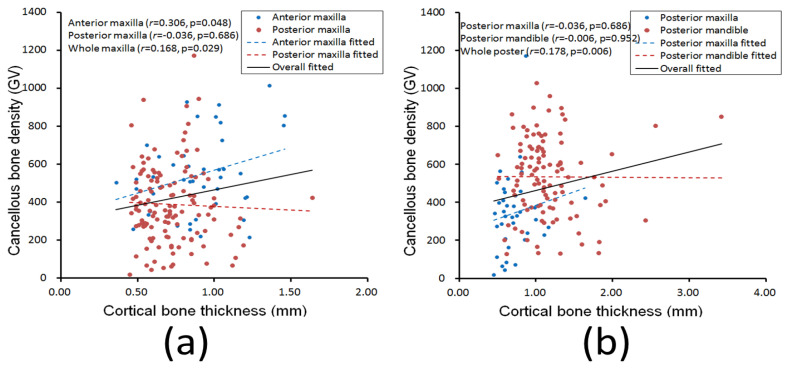
Relationships between cortical bone thickness and cancellous bone density at dental implant sites in the (**a**) anterior maxilla (blue dots and dotted line), posterior maxilla (red dots and dotted line), and the entire maxilla region (black line) and in the (**b**) posterior maxilla (blue dots and dotted line), posterior mandible (red dots and dotted line), and entire posterior region (black line).

The experimental results of this study revealed that crestal cortical bone thickness and cancellous bone density had different rankings at dental implant sites in the four jawbone regions. The posterior maxilla had the lowest cancellous bone density and the thinnest crestal cortical bone. However, no correlation was observed between the crestal cortical bone thickness and cancellous bone density in this region (*p* > 0.05). Figure 5a presents scatter plots of cortical bone thickness and cancellous bone density of the anterior maxilla and posterior maxilla, respectively, of all patients. The two indicators were not correlated in the posterior maxilla (*p* > 0.05); however, because cancellous bone density and crestal cortical bone thickness in the posterior maxilla were much lower than those in the anterior maxilla, the two indicators were weakly correlated in the maxilla region (*r* = 0.168, *p* = 0.029). Similarly, the two indicators were not correlated in the posterior maxilla or posterior mandible alone (*p* > 0.05), but the overall values in the posterior maxilla were lower than those in the posterior mandible. Therefore, the two indicators demonstrated a weak correlation in the posterior region (*r* = 0.178, *p* = 0.006, Figure 5b). 

Numerous studies have verified that cancellous bone density and crestal cortical bone thickness at dental implant sites greatly influence the success rate of dental implant surgery [3,5,6,7,21,22,27] because dental implants with superior bone conditions have greater initial stability [3,5,6,7]. However, cortical and cancellous bone have different roles in maintaining the stability of dental implants [22,27]. Cortical bone is generally related to the initial stability of dental implants, whereas cancellous bone, which is composed of trabecular bone and is filled with more blood, is more related to osseointegration in subsequent stages. The most common clinical approach for assessing bone quality and quantity in the jaw is the use of the Lekholm and Zarb bone classification [28]. Jawbones are categorized into four types from most to least preferable, and this classification method was established on the basis of the premise that thicker cortical bone results in denser cancellous bone, whereas thinner cortical bone contributes to looser cancellous bone. Nevertheless, this study revealed that crestal cortical bone thickness and cancellous bone density differed inconsistently at the dental implant sites in the four jawbone regions; furthermore, cancellous bone density and cortical bone thickness were weakly correlated and even exhibited no correlation in some regions. This suggests that thicker cortical bone is not necessarily associated with denser cancellous bone. Accordingly, for patients requiring dental implants, dentists can assess the cortical and cancellous bone conditions at implant sites by using dental CBCT to develop comprehensive preoperative plans for dental implants. 

This study had the following limitations: (1) All patients recruited in this study were Asian; whether these results are generalizable to other ethnic groups requires further examination. (2) Patients in this study were not grouped by sex or age because the sample size was insufficient. (3) Cancellous bone density was measured as radiographic density (in GVs) in this study. However, several researchers have indicated that dental CBCT is not as accurate as clinical CT is for measuring bone density. In the future, a calibration phantom should be employed alongside CBCT for calculations of bone mineral density at a dental implant site. (4) The patients selected in this study were carefully evaluated by the dentist and judged to be suitable candidates for dental implant surgery. This study did not include the effects of specific patients’ conditions (e.g., how long patients used removable prostheses, time from tooth extraction, and patients’ physical conditions). (5) This study examined only patients’ dental CBCT images prior to their dental implant surgery and did not perform follow-ups to determine survival rates after surgery. In subsequent research, we plan to implement more comprehensive assessments of the influence of jawbone condition on dental implant survival rates.

## 5. Conclusions

The following conclusions regarding host bone condition at the dental implant site in the four jawbone regions were drawn on the basis of the experimental setup and limitations:

(1) Cancellous bone density was highest in the anterior mandible (722 ± 227 GV), followed by the anterior maxilla (542 ± 208 GV), posterior mandible (535 ± 206 GV), and posterior maxilla (388 ± 206 GV).
(2) The cortical bone was thickest in the posterior mandible (1.15 ± 0.42 mm), followed by the anterior mandible (1.01 ± 0.32 mm), anterior maxilla (0.89 ± 0.26 mm), and posterior maxilla (0.72 ± 0.19 mm).
(3) A weak correlation (*r* = 0.133, *p* = 0.041) was observed between cancellous bone density and cortical bone thickness in the entire jawbone. Furthermore, except for the anterior maxilla (*r* = 0.306, *p* = 0.048), no correlation between the two bone parameters was detected.

## Figures and Tables

**Table 1:** Relationship between cancellous bone density and cortical bone thickness at dental implant sites.

Region of the Jawbone	Numbered	Cancellous Bone Density (Mean ± SD)	Cortical Bone Thickness (Mean ± SD)	Pearson Correlation Coefficient	
				*r*	*p*
All region	315	500 ± 235	0.92 ± 0.36	0.133	0.041
Mandible	146	585 ± 227	1.11 ± 0.40	−0.125	0.133
Maxilla	169	426 ± 217	0.76 ± 0.23	0.168	0.029
Anterior	81	629 ± 235	0.95 ± 0.30	0.049	0.662
Posterior	234	456 ± 219	0.92 ± 0.38	0.178	0.006
Anterior maxilla	42	542 ± 208	0.89 ± 0.26	0.306	0.048
Anterior mandible	39	722 ± 227	1.01 ± 0.32	−0.296	0.068
Posterior maxilla	127	388 ± 206	0.72 ± 0.19	−0.036	0.686
Posterior mandible	107	535 ± 206	1.15 ± 0.42	−0.006	0.952
Unit		GV (Grayscale value) mm			
